# An End-to-End Particle Gradation Detection Method for Earth–Rockfill Dams from Images Using an Enhanced YOLOv8-Seg Model

**DOI:** 10.3390/s25154797

**Published:** 2025-08-04

**Authors:** Yu Tang, Shixiang Zhao, Hui Qin, Pan Ming, Tianxing Fang, Jinyuan Zeng

**Affiliations:** 1 School of Infrastructure Engineering, Dalian University of Technology, Dalian 116024, China; ytang@dlut.edu.cn (Y.T.);; 2 Nanjing Hydraulic Research Institute, Nanjing 210029, China; pming@nhri.cn

**Keywords:** earth–rockfill dam, particle gradation detection (PGD), image instance segmentation (IIS), dual-attention mechanism, transfer learning

## Abstract

Rockfill particle gradation significantly influences mechanical performance in earth–rockfill dam construction, yet on-site screening is often time-consuming, labor-intensive, and structurally invasive. This study proposes a rapid and non-destructive detection method using mobile-based photography and an end-to-end image segmentation approach. An enhanced YOLOv8-seg model with an integrated dual-attention mechanism was pre-trained on laboratory images to accurately segment densely stacked particles. Transfer learning was then employed to retrain the model using a limited number of on-site images, achieving high segmentation accuracy. The proposed model attains a mAP50 of 97.8% (base dataset) and 96.1% (on-site dataset), enabling precise segmentation of adhered and overlapped particles with various sizes. A Minimum Area Rectangle algorithm was introduced to compute the gradation, closely matching the results from manual screening. This method significantly contributes to the automation of construction workflows, cutting labor costs, minimizing structural disruption, and ensuring reliable measurement quality in earth–rockfill dam projects.

## 1. Introduction

Earth–rockfill dams are one of the most widely used hydraulic structures due to their adaptability and the use of local materials. The particle gradation of rockfill materials plays an important role in determining the compaction quality, permeability resistance, and overall strength and stability of the dam [1,2,3]. To ensure high-quality compacting construction of structures, adopting a reasonable particle gradation distribution is indeed crucial [4,5,6]. The basic task is to conduct the particle gradation detection (PGD) quickly and accurately at construction sites. The on-site screening test is a traditional PGD method, in which random samples are extracted for manual or mechanical sieving, and then particle grading curves are obtained by statistical analysis [7,8]. Although this method is technically mature, it requires considerable time and labor, and it may destroy the integrity of the structure to some extent as well. In addition, it has great limitations in terms of low sampling, which can only represent the particle sizes of a certain point, resulting in unsmooth and incomplete particle grading curves [9]. Therefore, it is of great importance to develop an efficient and accurate PGD method for a massive amount of filling materials in earth–rockfill dam construction.

Computer vision (CV) has been extensively applied for image segmentation to extract morphological characteristics of objects, making rapid on-site detection of particle gradation possible [10,11]. Digital image processing (DIP) of image segmentation, such as edge segmentation [12,13], threshold segmentation [14,15,16] and morphology-based segmentation [17,18,19], is widely used for the extraction of particle geometry and contours. Guo et al. [20] investigated the stereo-logical estimation of aggregate gradation for asphalt mixtures based on a morphology-based method. By assuming the aggregate as an ellipsoidal particle in stereo-logical conversion, the gradation can be accurately estimated with a small number of images. Yu et al. [21] designed an image-recognition-based gradation detection system for earth and rock materials. Threshold methods are utilized for image segmentation, and particle contours are detected through the edge detection algorithm of Canny to recognize particle sizes. The system allows the rapid and accurate detection of gradation at a dam construction site. Wang et al. [22] developed a modified watershed-based segmentation method to assess particle size and morphology distributions of aggregate particles. It is verified that this method can accurately extract the contour characteristics of contacting coarse particles, resulting in rapid image processing. Traditional DIP-based gradation detection methods can efficiently recognize aggregate particle contours and extract their characteristics. However, the recognition accuracy is highly dependent on the manually selected segmentation algorithm parameters. Inappropriate parameters in these methods will lead to under- or over-segmentation of the objects.

With the enhancement of computing power and the boosting of algorithms, deep learning (DL) has made a breakthrough in the field of image segmentation, especially image instance segmentation (IIS). Unlike traditional segmentation methods, IIS offers a detailed recognition of object instances at the pixel level and enhances the possibility of high-resolution feature extraction of the objects (i.e., sizes, shapes, edges, etc.) [23,24,25,26,27,28]. In the last decade, a variety of IIS models have been developed, such as Mask2Former [29], Mask R-CNN [30], SOLOv2 [31], and YOLACT++ [32]. Among the others, Mask R-CNN achieves distinguished performance on extracting pixel-level features of particles with ResNet as the backbone, and realizes top-down instance segmentation based on a region proposal network and mask prediction heads [33,34,35,36]. Zhang et al. [37] established a sand-like granular matter characterization procedure based on Mask R-CNN, achieving satisfying performance on segmenting contacted, overlapped, or even densely packed sand particles. Li et al. [38] introduced atrous convolution into Mask R-CNN to extract the feature characteristics of pulverized coal particles with relatively high accuracy, achieving end-to-end segmentation with no pre-processing and post-processing operations. While Mask R-CNN offers significant advantages in instance segmentation and good multi-scale adaptability, the model’s high computational demands and long training duration become bottlenecks. This hinders its use in rapid rockfill material segmentation within complex scenarios. In addition, the segmentation of small and overlapped particles based on Mask R-CNN remains a challenge.

In 2023, the YOLOv8 framework was proposed by Ultralytics, which is a variant of the You Only Look Once (YOLO) family, well known for their real-time processing capabilities and outstanding performance of object detection [39,40,41,42,43,44,45,46]. YOLOv8-seg is an extended edition of the YOLOv8 series, specifically designed for instance segmentation [47]. It shows a great advantage in various scenarios that require rapid and fine object segmentation, such as medical imaging [48], autopilot [49] and precision agriculture [50]. Compared to Mask R-CNN, YOLOv8-seg is more lightweight, faster in inference, and has lower hardware requirements. In recent years, some researchers have tried to advance the applications of YOLOv8-seg in the field of civil and hydraulic engineering, such as crack detection [51,52] and construction monitoring [53,54]. For particle instance segmentation, Hu et al. [55] developed an evaluation method for the spatial uniformity and blending ratio consistency of continuous gravelly soil based on an improved YOLOv8-seg model. They modified the original model by employing a conditional convolution segmentation head and an improved Protonet to enhance the segmentation accuracy with less computation cost. It is worth noting that YOLOv8-seg still uses the segmentation headers of YOLACT, which generates the final segmentation mask by predicting a shared set of segmentation prototypes and the coefficients of each instance. Accurately segmenting adhered and overlapped objects is challenging due to the difficulty in generating individual masks per object. Consequently, both segmentation accuracy and model robustness may compromise.

Another critical issue in the application of image-based PGD lies in accurately estimating particle volumes from 2D images. In general, the estimation of particle volumes is based on the assumption that the particle shape can be approximated to be a prolate spheroid [56], and the equivalent diameters of 2D particle projection in the form of an ellipse are adapted for the division of particle size intervals. The Feret diameter method is a commonly used representation of the projected size of particles. In recent years, it has been successfully applied to calculate the major and minor diameters of ellipses to estimate the particle volume for PGD, and good results can be obtained when there is no obstruction or only minor occlusion [57,58]. However, significant calculation errors arise when particles exhibit excessive overlap or occlusion. Precise diameter determination is hindered by fragmented contour data. The Minimum Area Rectangle (MAR) algorithm is another widely used approach in geometry, spatial analysis, automated driving and geographic information systems (GISs), which encloses a set of points or an object within the smallest possible rectangle [59,60]. Since the MAR algorithm fits the boundary of the object’s contour, it does not rely on the regularity or completeness of the object’s shape. Even if the object is partially obscured, the unobscured portion of the contour can still effectively reflect the approximate shape of the object, allowing the geometrical features of the object to be represented. So far, studies applying the MAR algorithm in particle size calculation are relatively scarce. Fan et al. [61] tried to estimate the volume of gravel particles based on the MAR algorithm by calculating the long and short axes of the Minimum Area Rectangle. The results showed that the algorithm is able to calculate the particle size and gradation. However, the computational accuracy and robustness under conditions where particles are excessively obscured need further investigation.

To address the challenges of particle size variation, mutual occlusion, and overlapping in densely packed rockfill materials, an IIS-based PGD method was proposed in this study. The method captures field images, segments particle contours using a modified YOLOv8-seg model, and calculates gradation with the MAR algorithm. Transfer learning is employed to address limited on-site data, and morphological features are extracted via a rotation-based MAR strategy. The main contributions are: (1) integrating portable imaging and deep learning for real-time particle segmentation and gradation calculation; (2) enhancing feature focus and small-target accuracy using CBAM and SE blocks; (3) leveraging transfer learning for robust segmentation under small-sample conditions; and (4) achieving reliable volume and gradation classification despite particle occlusion. 

The remainder of this paper is organized as follows: Section 2 discusses the fundamental principles of the approach, including data acquisition and pre-processing, particle segmentation and gradation detection. Section 3 conducts model training and assesses the segmentation performance of the model, Section 4 presents an experimental study to validate the proposed methodology, Section 5 discusses the impact of transfer learning on the segmentation performance of the model, and Section 6 summarizes the main findings of this work.

## 2. Methodology

**Figure 1:**
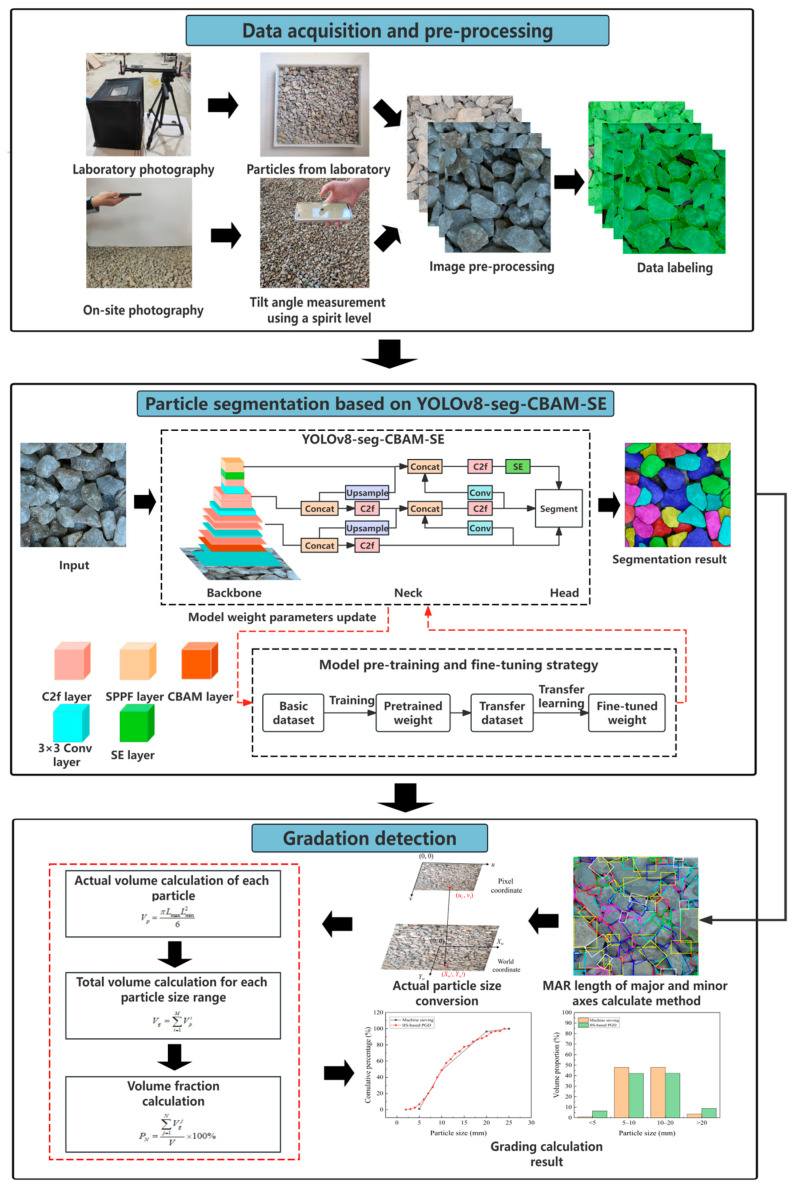
Framework of the IIS-based PGD method.

The framework of the IIS-based PGD method is shown in Figure 1, consisting of three steps: (1) data acquisition and pre-processing, (2) particle segmentation based on YOLOv8-seg-CBAM-SE, and (3) gradation detection. In the first step, two different datasets of particle images are established by using a photography setup with a high-resolution camera in the laboratory and a portable camera device (e.g., smartphone) at construction sites, respectively. In the second step, the particles are segmented at the pixel level by YOLOv8-seg-CBAM-SE, and transfer learning is employed to enhance the segmentation performance of the model for on-site images. In the third step, the MAR algorithm with a revolving angle strategy is employed to calculate the gradation of particles.

### 2.1. Data Acquisition and Pre-Processing

Due to the complex environments at construction sites, the quality of on-site particle images is greatly affected by the lighting condition, shooting distance and tilt angle. Additionally, it is difficult to collect a sufficient amount of data for DL-based IIS in a short time, which is not conducive to the rapid gradation detection of rockfill materials. To solve these issues, a large amount of particle images is collected in the laboratory for pre-training of the IIS model, and on-site particle images are obtained for retraining to fine-tune the parameters of the model.

#### 2.1.1. Image Acquisition

**Figure 2:**
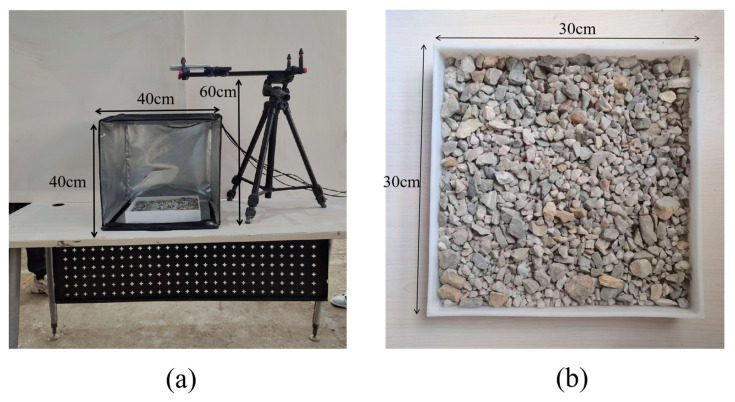
Laboratory photography: (**a**) controlled photography setup; (**b**) image captured by the camera.

In this study, a controlled photography setup was established in the laboratory to capture particle images. The setup consists of a blackout box, a polypropylene tray, a high-resolution camera, and a retractable mount, as illustrated in Figure 2a. The dimensions of the blackout box are 40 cm × 40 cm × 40 cm, while the tray measures 30 cm × 30 cm × 5 cm. The camera is securely mounted on the retractable stand and positioned directly above the tray at a distance of 60 cm. Then, images are captured for dispersed and densely stacked particles in varied background colors, sizes and shapes, as shown in Figure 2b.

**Figure 3:**
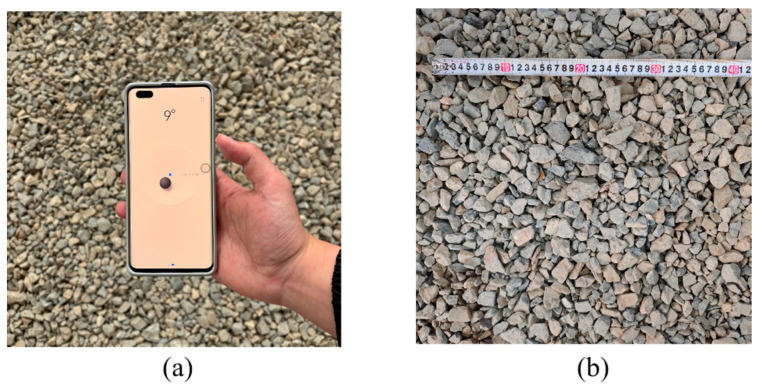
On-site photography: (**a**) images captured by a smartphone; (**b**) image calibration.

For on-site image collection, photographs are taken using a smartphone positioned parallel to the objects. A leveling application on the smartphone is employed to measure and maintain a consistent tilt angle during image capture, as shown in Figure 3a. The smartphone is then positioned above the objects for photography, with the shooting distance adjustable for different particle sizes. To facilitate the conversion of pixel dimensions into real-world measurements, a calibration scale is placed within the test area, as shown in Figure 3b.

#### 2.1.2. Image Pre-Processing

(1) Denoising: The images are first pre-processed using the Opening operation with a 3 × 3 convolution kernel for denoising. The Opening is a mathematical morphology technology that quantitatively characterizes geometric structures based on set theory, which consists of an erosion operation followed by a dilation operation [62]. During erosion, the input image (Im) is scanned using a structuring element (Se), removing foreground pixels that do not conform to its shape. Subsequently, dilation is applied to restore the primary structure of larger connected regions while preventing the reappearance of small noise artifacts. The Opening of Im by Se is formally defined as

(1) Im∘Se=ImΘSe⊕Se,

where Θ and ⊕ denote the erosion and dilation operations, respectively.

**Figure 4:**
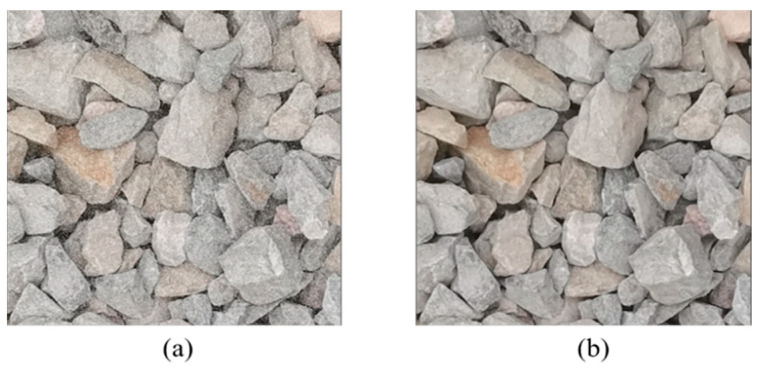
An example particle image: (**a**) before denoising; (**b**) after denoising.

To assess the denoising performance of the Opening operation, a 640 × 640 particle image was corrupted with pepper noise at a noise density of 0.15 and then processed based on Equation (1), as shown in Figure 4. It can be seen that the noise is effectively removed while the integrity of edge features is maintained. Furthermore, a quantitative evaluation was carried out by computing the signal-to-noise ratio (SNR) values. Specifically, the SNR of the pre-denoising image was measured to be 12.27 dB, whereas that of the post-denoising image increased to 25.82 dB, demonstrating the remarkable denoising performance of the Opening operation.

**Figure 5:**
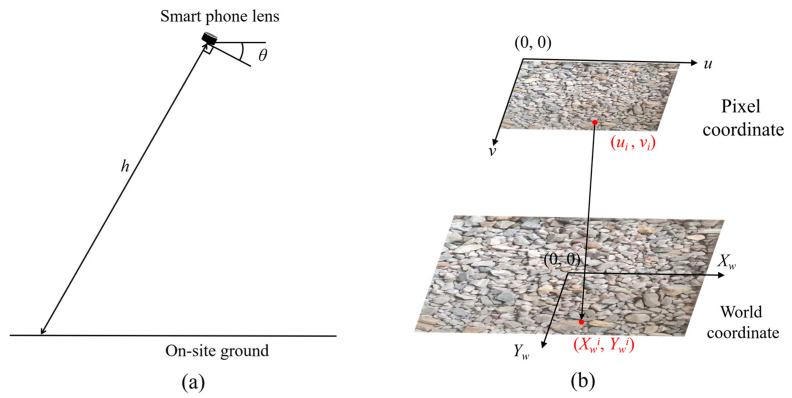
Coordinate transformation: (**a**) on-site photography; (**b**) mapping of the pixel coordinate to the world coordinate.

(2) Coordinate transformation: Image distortion results in a discrepancy between the particle sizes depicted in the image and their actual physical dimensions, leading to non-proportional scaling. To address this issue, a coordinate transformation is implemented to establish a precise relationship between the pixel coordinate and the world coordinate. This transformation incorporates critical parameters such as the shooting distance h, tilt angle θ, pixel coordinate (u, v) and world coordinate (*X_W_*, *Y_W_*), as illustrated in Figure 5.

Based on the perspective projection theory and rigid body transformation theory, the relationship between the pixel and world coordinates for the *i*-th point can be expressed as [63]

(2) $$s\left[\begin{array}{c} \begin{array}{c} u_{i}\\ v_{i} \end{array}\\ 1 \end{array}\right]=K\left[\begin{array}{cc} R & t \end{array}\right]\cdot \left[\begin{array}{c} X_{w}^{i}\\ Y_{w}^{i}\\ Z_{w}^{i}\\ 1 \end{array}\right],$$

(3) $$K=\left[\begin{array}{ccc} f_{x} & 0 & u_{0}\\ 0 & f_{y} & v_{0}\\ 0 & 0 & 1 \end{array}\right],$$

(4) $$R=\left[\begin{array}{ccc} 1 & 0 & 0\\ 0 & \cos\theta & -\sin\theta \\ 0 & \sin\theta & \cos\theta \end{array}\right],$$

(5) $$t=\left[\begin{array}{c} \begin{array}{c} 0\\ -h\sin\theta \end{array}\\ h\cos\theta \end{array}\right],$$

where *s* is the homogeneous scaling factor; **K** is the intrinsic matrix of the camera; **R** and **t** are the extrinsic parameters of the camera denoting rotation and translation, respectively; $Z_{w}^{i}$ is the world coordinate of *i*-th point in the *Z_w_*-direction, and it equals zero when ignoring the depth information of the object; *f_x_* and *f_y_* are the focal lengths (in meters), respectively; *u*_0_ and *v*_0_ are the coordinates of the principal point (in pixels), respectively.

Substituting Equations (3)–(5) into Equation (2), we obtain

(6) $$s\left[\begin{array}{c} u_{i}\\ v_{i}\\ 1 \end{array}\right]=\left[\begin{array}{c} f_{x}x_{i}+u_{0}(y_{i}\sin\theta +h\cos\theta )\\ f_{y}(y_{i}\cos\theta -h\sin\theta )+v_{0}(y_{i}\sin\theta +h\cos\theta )\\ y_{i}\sin\theta +h\cos\theta \end{array}\right],$$

then,

(7) $$s=Y_{w}^{i}\sin\theta +h\cos\theta ,$$

Substituting Equation (7) into Equation (6), the transformation from the pixel coordinate to the world coordinate can be established as follows:

(8) $$\left\{\begin{array}{l} Y_{w}^{i}=\frac{-h\left[f_{y}\sin\theta +(v_{i}-v_{0})\cos\theta \right]}{\left(v_{i}-v_{0}\right)\sin\theta -f_{y}\cos\theta }\\ X_{w}^{i}=\frac{\left(u_{i}-u_{0})(y_{i}\sin\theta +h\cos\theta \right)}{f_{x}} \end{array}\right.,$$

**Figure 6:**
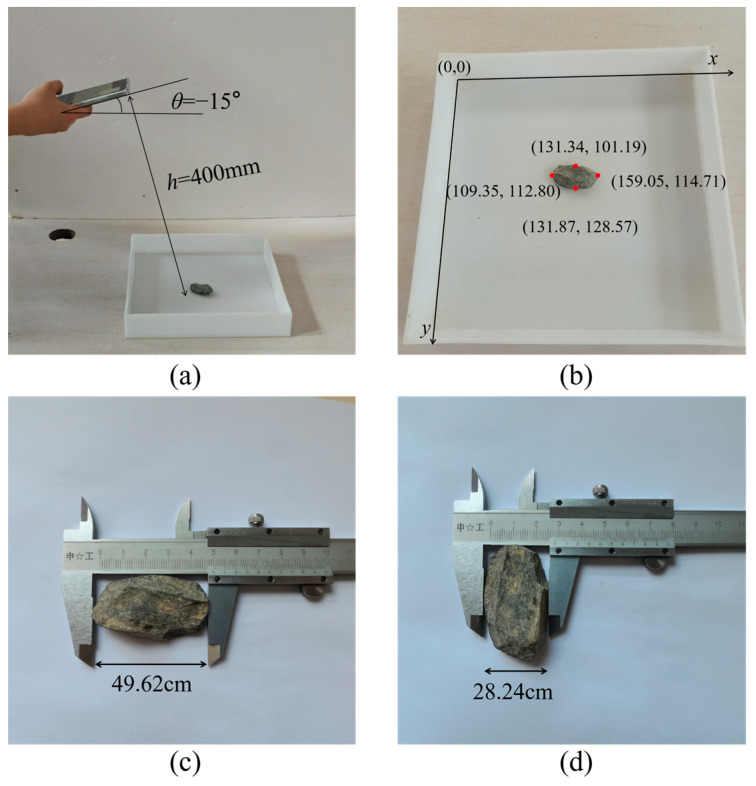
Validation of coordinate transformation: (**a**) photography; (**b**) pixel coordinate; (**c**) *L*_max_ in the world coordinate; (**d**) *L*_min_ in the world coordinate.

To verify Equation (8), an experimental test was designed and executed in the laboratory. Specifically, a particle was randomly positioned on the tray and captured using a smartphone at a tilt angle of *θ* = −15° and a height of *h* = 40 cm, as depicted in Figure 6a. Subsequently, the pixel coordinates of the four key points, which define the major axis length (*L*_max_) and minor axis length (*L*_min_) of the particle, were precisely measured and are presented in Figure 6b. The measured values of *L*_max_ and *L*_min_ are 49.73 mm and 27.39 mm, respectively, while the corresponding actual lengths obtained through direct physical measurement are 49.62 mm and 28.24 mm, as shown in Figure 6c,d. The calculated mean absolute error (MAE) of 1.61% indicates that the coordinate transformation in Equation (8) exhibits high accuracy and satisfies the stringent requirements of on-site measurement applications.

#### 2.1.3. Data Labeling

Data labeling is a critical component in supervised machine learning, as the quality of the training data directly impacts the performance of the resulting model. Conventionally, data labeling is a labor-intensive process that predominantly depends on manual annotation. In particular, for particles that are adhered and overlapped, it is extremely challenging to precisely identify the boundaries with the naked eye. To address this problem, we adopt ISAT-SAM (Interactive Segment Anything Tool–Segment Anything Model) to conduct labeling for the particle images obtained both in the laboratory and at the construction site. The annotation process comprises three steps:

(1) Automatic segmentation: SAM is employed to automatically segment the input image, generating initial segmentation masks and thereby substantially alleviating the manual annotation workload.

(2) Interactive optimization: In cases where the automatic segmentation results are incomplete or lack precision, the initial segmentation masks can be manually refined using ISAT, enabling modifications such as the addition, deletion, or adjustment of segmentation boundaries.

(3) Iterative optimization: Iteratively alternate between automatic segmentation and manual adjustments until satisfactory segmentation results are achieved.

**Figure 7:**
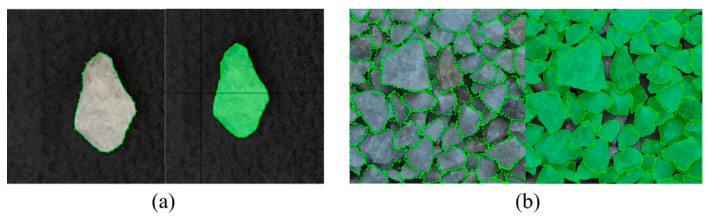
Data labeling: (**a**) single particle; (**b**) densely stacked particles.

Following the procedure above, particles with varying sizes, background colors, and stack conditions are labeled, as shown in Figure 7. Thus, two sets of data, including particle images collected in the laboratory (named as “Base dataset”) and the ones obtained on-site (named as “On-site dataset”), are prepared and used for model training.

### 2.2. Particle Segmentation Based on YOLOv8-Seg-CBAM-SE

#### 2.2.1. Overview of YOLOv8-Seg

YOLOv8-seg integrates object detection with semantic segmentation in a state-of-the-art framework. Through multi-scale feature fusion and optimized strategies, it delivers efficient, high-precision target recognition and segmentation. However, the YOLO architecture inherently struggles with small-scale objects, which often exhibit limited pixel representation in images. Additionally, the segmentation head of YOLOv8-seg relies on convolutional operations on local features to produce segmentation masks. In cases of overlapped or blurred boundaries, these local features may fail to capture subtle inter-object distinctions, resulting in merged pixels from different targets and inaccurate segmentation contours.

#### 2.2.2. YOLOV8-Seg-CBAM-SE

**Figure 8:**
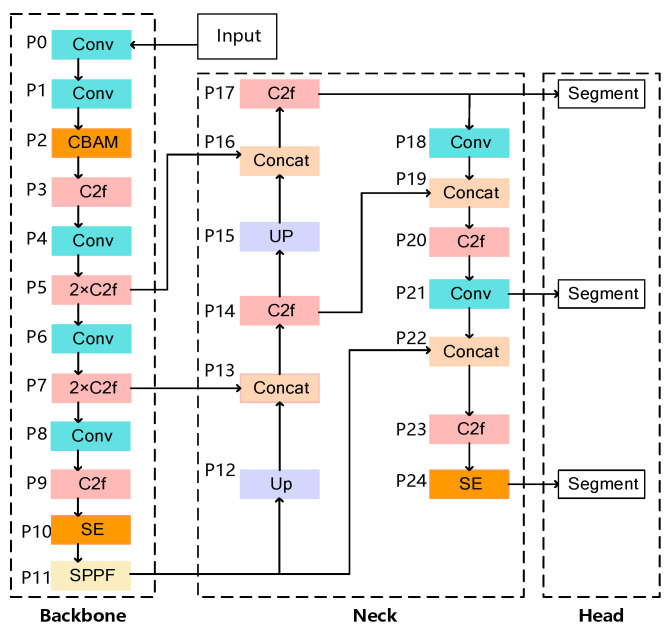
The architecture of YOLOv8-seg-CBAM-SE.

A dual-attention model, named YOLOv8-seg-CBAM-SE, is designed to enhance particle segmentation accuracy in cluttered construction environments, where overlapping or blurred particles pose significant challenges. The proposed model integrates two advanced attention mechanisms, i.e., the convolutional block attention module (CBAM) and SE block, into the framework. The overall architecture of the proposed model is comprised of three distinct components, backbone, neck, and head, as shown in Figure 8.

(1) Backbone: In the feature extraction stage, the model performs initial feature extraction through multi-layer convolutional operations (Conv), subsequently incorporating the CBAM to enhance discriminative feature representation through dual-channel and spatial attention mechanisms. The cross-stage partial fusion (C2f) module then conducts cross-resolution feature fusion, preserving multi-scale structural details critical for particle boundary delineation in cluttered environments.

(2) Neck: During the multi-level feature fusion stage, the model hierarchically integrates multi-scale features through upsampling and feature concatenation. Sequentially deployed SE blocks perform channel-wise recalibration to amplify salient features, while C2f modules enable inter-scale context propagation. This architecture systematically optimizes saliency representation and boundary discrimination accuracy, particularly for occluded particles in construction debris scenarios.

(3) Head: In the segmentation head stage, the architecture executes dual tasks through coordinated components: (1) the detection head coordinates target localization and categorical identification, while (2) the mask generator precisely reconstructs per-instance segmentation masks through prototype-based decoding. These components are synergistically connected via shared mask prototypes and hierarchical feature propagation, achieving simultaneous particle detection and geometric contour segmentation for PGD.

**Figure 9:**
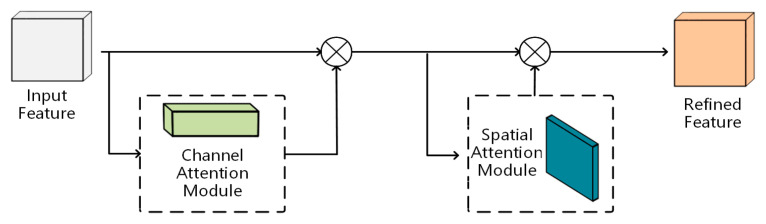
Convolutional block attention module (CBAM).

CBAM [64] constitutes a lightweight convolutional attention mechanism that operates through sequential channel–spatial recalibration. As a plug-and-play component, it refines intermediate features by simultaneously modeling channel-wise dependencies and spatial correlations through learnable attention weights, effectively amplifying discriminative features while attenuating background interference. Integrated into the backbone network, this dual-attention module significantly augments the network’s contextual perception capability for particle contour characterization in cluttered construction environments and achieves enhanced segmentation accuracy, as seen in Figure 9.

**Figure 10:**
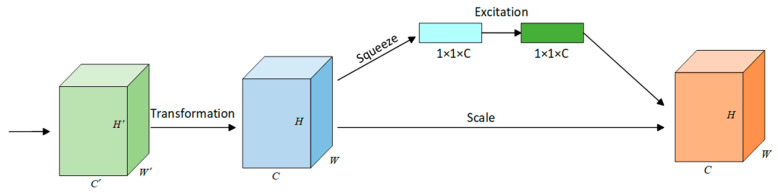
Squeeze and excitation block (SE).

The SE block [65] operates through three consecutive transformations: (1) squeeze aggregates global channel statistics via global average pooling, (2) excitation estimates channel-wise interdependencies through gated sigmoid activations, and (3) scale recalibrates feature responses by channel-wise multiplication, as shown in Figure 10. Integrated into both the backbone and neck layers of YOLOv8-seg, this gating mechanism dynamically prioritizes task-relevant features through global context modeling while suppressing spatial redundancy. Notably, its lightweight architecture introduces minimal parametric overhead, maintaining computational efficiency critical for real-time construction site deployment.

#### 2.2.3. Model Pre-Training and Fine-Tuning

**Figure 11:**
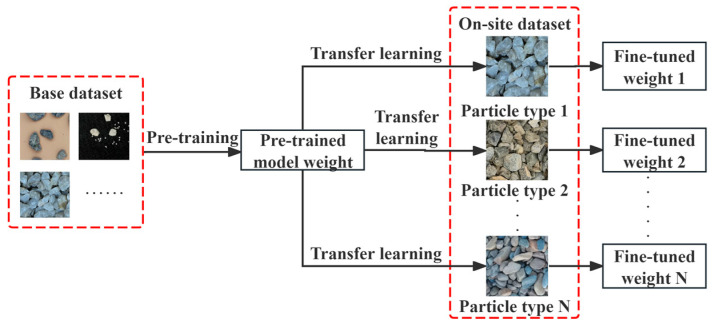
Model pre-training and fine-tuning strategy.

The proposed YOLOv8-seg-CBAM-SE adopts a two-phase training paradigm: (1) pre-training on a large-scale base dataset to establish domain-agnostic feature representations, followed by (2) fine-tuning using the on-site dataset, as shown in Figure 11. The on-site dataset employs a limited number of particle images captured under earth–rockfill dam conditions. This constrained sample regime enables few-shot adaptation of pre-trained weights for precise particle characterization, bridging the model adaptation gap in conventional deep learning approaches that suffer from inter-particle morphological variance sensitivity. Meanwhile, the lightweight architecture ensures efficient transfer learning scalability with minimal image samples, allowing rapid development of particle-specific on-site datasets for tailored model adaptation.

#### 2.2.4. Model Evaluation Criteria

The model’s performance in object detection and image segmentation is investigated through a comprehensive evaluation employing quantitative metrics including precision (P), recall (R), and mean average precision (mAP). The analysis specifically examines the system’s capability to accurately identify targets while minimizing false positives, as well as its effectiveness in delineating object boundaries during segmentation tasks.

The computation of precision and recall metrics relies on the confusion matrix framework, comprising three fundamental components: true positives (*TP*), false positives (*FP*), and false negatives (*FN*). *TP* refers to cases where the model correctly identifies positive class instances that are actually positive; *FP* denotes instances where the model incorrectly classifies a negative example as positive. *FN* refers to instances where the model fails to identify a positive example present in the ground truth. The corresponding formulas can be expressed as

(9) $$P=\frac{TP}{TP+FP},$$

(10) $$R=\frac{TP}{TP+FN},$$

Intersection over Union (IoU) represents the ratio of the area of overlap between the model-generated region and the original annotated region, used to evaluate the accuracy of an object detector in machine learning, particularly in tasks like image segmentation and object detection. It is defined as

(11) $$\mathrm{IoU}=\frac{OA\cap MG}{OA\cup MG},$$

where *OA* represents the original annotated region; *MG* represents the model-generated region.

The mAP is calculated by averaging the area under the precision–recall (PR) curve for each category as follows:

(12) $$\mathrm{mAP}=\frac{\int_{0}^{1}P(R)d(R)}{N},$$

where *N* is the number of object categories.

The mAP at the 50% IoU threshold (i.e., mAP50) represents a standard detection metric calculated as the mean average precision when using a single IoU threshold of 50%. In contrast, the mAP50-95 metric systematically varies the localization strictness from 50% to 95% in 5% increments (i.e., IoU = 0.50, 0.55, …, 0.95), thereby assessing model robustness under different detection precision requirements. The mAP50-95 can be expressed as

(13) $$\mathrm{mAP}50\text{-}95=\frac{1}{10}\sum_{k=0}^{9}\mathrm{mA}P_{\mathrm{IoU}=0.5+0.05k},$$

### 2.3. Particle Gradation Detection

**Figure 12:**
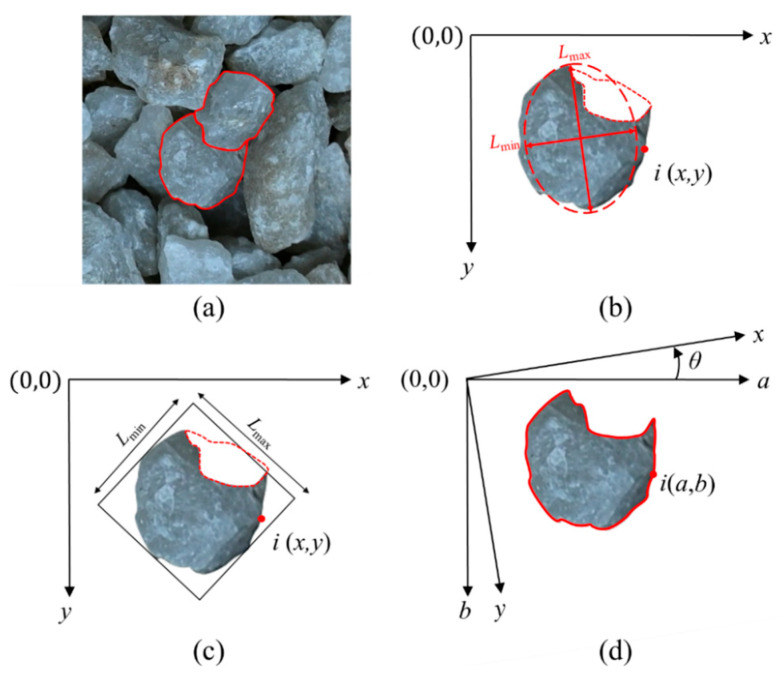
Particle size calculation: (**a**) partially obscured particles; (**b**) major and minor axes of the Feret method; (**c**) major and minor axes of the MAR algorithm; (**d**) particle contour points coordinate rotation.

The YOLOv8-seg-CBAM-SE architecture outputs instance segmentation results represented as polygonal contour coordinates, which serve as the geometric basis for particle size distribution analysis, as demonstrated in Figure 12a. For instance-segmented 2D particle imagery, we implement an ellipsoid spheroid approximation method for volumetric estimation, adopting the volume fraction to derive gradation characteristics. The measurement precision fundamentally relies on accurate determination of critical morphological parameters, specifically the major and minor axes of the fitted ellipsoids.

The most widely used method for determining major and minor axes is the Feret diameter method. Based on this method, *L*_max_ is derived from particle contours generated through instance segmentation, while *L*_min_ is estimated via the equivalent ellipse approximation method, ensuring robust measurements for isolated particles. As depicted in Figure 12b, partial occlusion of a particle reduces its equivalent elliptical area compared to the non-occluded state, leading to a significantly lower value of *L*_min_. Nevertheless, the classification of particles into gradation intervals is highly dependent on *L*_min_. Unfortunately, the Feret diameter method is prone to systematic errors, which cause particles to be misclassified into finer gradation categories than what their true sizes would warrant. In contrast, the MAR algorithm bypasses the computation of a particle’s projected area by focusing solely on the geometric extrema of its contour, as shown in Figure 12c [66].

**Figure 13:**
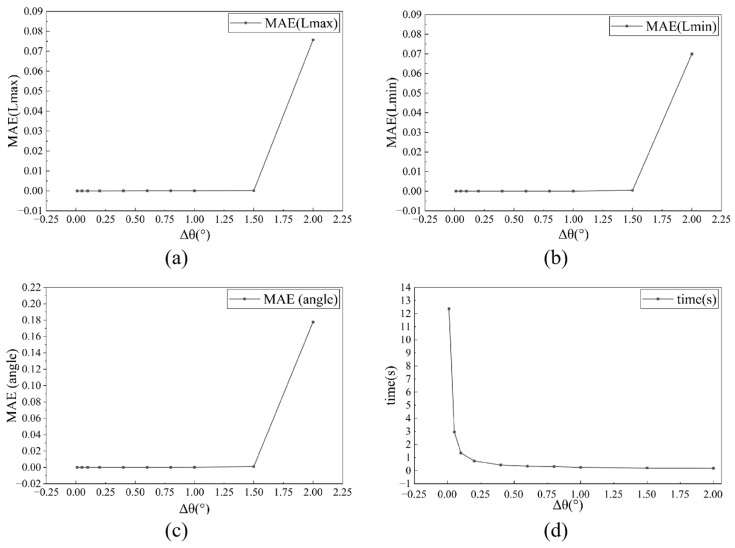
Relationship between angular resolution and MAR prediction accuracy/computational time: (**a**) MAR major axis as function of angular resolution (Δθ); (**b**) MAR minor axis as function of angular resolution (Δθ); (**c**) MAR rotation angle as function of angular resolution (Δθ); (**d**) computational time as function of angular resolution (Δθ).

Meanwhile, an angular discretization algorithm is employed to determine the MAR of planar geometric shapes. The algorithm incorporates coordinate system rotation to iteratively evaluate candidate circumscribed rectangles. Within the rotational range [0, π/2], the method performs angular discretization. It computes geometric projections at incremental angles and identifies the optimal MAR by comparing their enclosed areas. Figure 12d illustrates this process. To strike a balance between computational accuracy and efficiency, the angular parameter space denoted by θ is discretized utilizing a constant angular resolution where Δθ is set to 1° [67]. Figure 13a–c illustrate the variation of angular resolution (Δθ) with the mean absolute error (MAE) in terms of the major axis, minor axis, and rotation angle of the MAR, respectively. Figure 13d shows the variation of computational time per image with angular resolution. The results indicate that the MAR error undergoes a sharp increase when Δθ > 1°, while a sharp increase in computational time occurs when Δθ < 1°.

A systematic evaluation performed across all discretized angles facilitates the iterative computation of contour point coordinates when rotational transformations are applied, which is governed by the following two equations:

(14) $$\left\{\begin{array}{c} a=x\cos\theta_{i}-y\sin\theta_{i}\\ b=x\sin\theta_{i}+y\cos\theta_{i} \end{array}\right.,$$

where *a* and *b* denote the transformed coordinates following rotational operations.

When performing rotational transformations, the extremal coordinates (*a*_max_, *b*_max_) and (*a*_min_, *b*_min_) of the contour profiles can be derived through the following equations:

(15) $$\left\{\begin{array}{l} a_{\max}=\max(\{a_{1},a_{2},\ldots ,a_{n}\})\begin{array}{cc} , & a_{\min}=\min(\{a_{1},a_{2},\ldots ,a_{n}\}) \end{array}\\ b_{\max}=\max(\{b_{1},b_{2},\ldots ,b_{n}\})\begin{array}{cc} , & b_{\min}=\min(\{b_{1},b_{2},\ldots ,b_{n}\}) \end{array} \end{array}\right.,$$

where *n* is the total number of discretized contour points of a particle.

The area *S_i_* of the MAR corresponding to *θ_i_* is then computationally determined through extremal coordinate analysis:

(16) $$S_{i}=(a_{\max}-a_{\min})\cdot (b_{\max}-b_{\min}),$$

The global minimum area *S*_min_ and its corresponding optimal rotation angle *θ*_min_ are obtained via exhaustive search across all discretized angles *θ_i_*∈(0, π/2). For each *θ_i_*, the axis-aligned bounding box (AABB) dimensions are computed, from which the candidate area *S_i_* is derived. The parametric extremum is then identified through global comparison of {*S_i_*}. The determination of particle gradation in practical engineering applications relies on mechanical sieving using screens. Given the critical influence of the smallest particle dimension on the grading outcome, we represent a particle’s classification within the grading system by the *L*_min_ corresponding to its MAR. Subsequently, *L*_max_ and *L*_min_ of MAR are derived by

(17) $$\left\{\begin{array}{c} L_{\max}=\max(a_{\max}-a_{\min},b_{\max}-b_{\min})\\ L_{\min}=\min(a_{\max}-a_{\min},b_{\max}-b_{\min}) \end{array}\right.,$$

Assuming that individual particles approximate prolate ellipsoids, the volume of the individual particle *V_p_* can be expressed as

(18) $$V_{p}=\frac{\pi L_{\max}L_{\min}^{2}}{6},$$

The gradation characterization is quantified through volumetric cumulative distribution analysis across particle size intervals, substituting traditional mass-based quantification. Under the assumption of uniform particle density, the volume of the single interval *V_g_* is derived from

(19) $$V_{g}=\sum_{i=1}^{M}V_{p}^{i},$$

where *M* is the total number of particles in the interval.

The ratio of the cumulative volume of the *N*th gradation interval to the total volume *V* of the materials is expressed as

(20) $$P_{N}=\frac{\sum_{j=1}^{N}V_{g}^{j}}{V}\times 100\%,$$

in which *P_N_* represents the particle gradation cumulative fraction of the *N*th gradation interval.

## 3. Model Training and Testing

### 3.1. Dataset Establishment

**Figure 14:**
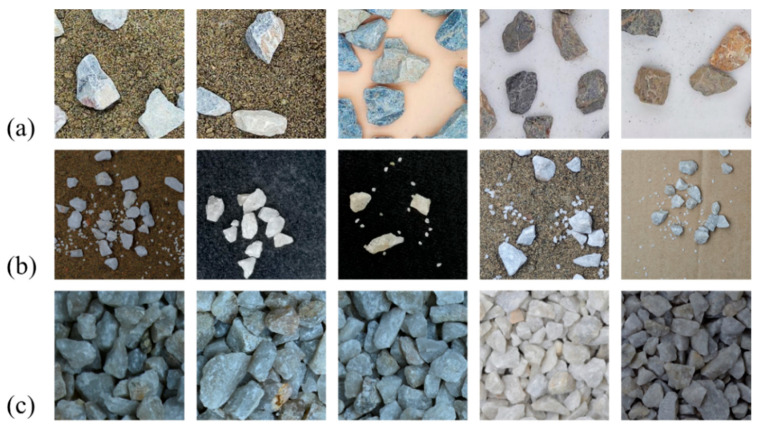
Base dataset: (**a**) Group I; (**b**) Group II; (**c**) Group III.

The particle images utilized for the dataset were acquired through both the laboratory and on-site photography methodologies, as detailed in the last section. Special attention was paid to ensure the variations in background colors, illumination conditions, particle sizes, particle colors and distribution states (i.e., sparse and stacked configurations). In particular, the base dataset is categorized into three groups based on the distribution states: (1) Group I: 459 sparse-distributed particle images (Figure 14a); (2) Group II: 760 partially overlapped particle images (Figure 14b); and (3) Group III: 2226 intensively overlapped particle images (Figure 14c). The light intensity was randomly modulated to mimic a wide variety of illumination conditions during the studio photography. Group I utilized basalt gravel particles, typically characterized by a coloration ranging from blue-black to dark gray. Their morphology is angular, with rough surfaces and irregular edges, which can significantly affect the way light is reflected and absorbed during imaging. Groups II and III utilized quartzite gravel particles, exhibiting a color spectrum from white to pale yellow. They possess sharp angularity, with shapes that are subregular and either blocky or flaky. The surfaces of these particles are smooth, and they feature prominent granular blastic textures.

**Figure 15:**
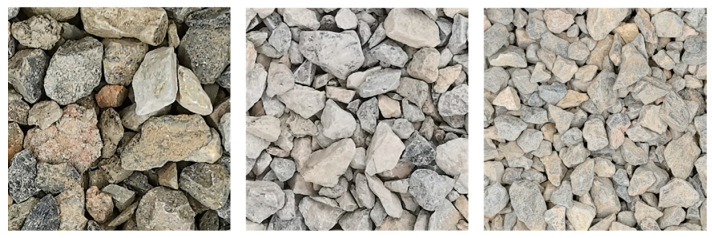
On-site dataset.

The on-site dataset consists of 150 images captured in the field. It is important to note that the particle shape and color differ from those obtained in the laboratory, as shown in Figure 15. The on-site dataset is primarily composed of limestone particles, which present light-colored hues. In terms of morphology, these particles manifest irregular blocky or flaky geometries. Unlike basalt particles, the edges of limestone particles are less sharply defined, and they feature relatively flat fracture surfaces.

Following the pre-processing and annotation stage, the dataset was partitioned into training, validation, and testing sets to ensure robust model development and evaluation. For model pre-training, the numbers of images used for each set are listed in Table 1. For model fine-tuning, the on-site dataset is divided into training and testing sets. The training set consists of 70 images to fine-tune the model’s weight parameters, while the testing set consists of 80 images to evaluate the model’s practical performance in real on-site construction.

### 3.2. Training Environment

In this study, all the models were trained on a Windows 10 operating system. The hardware environment consists of a 9th Gen Intel Core i9-9920x CPU@3.5 GHz, NVIDIA GeForce RTX 2080Ti 11 GB GPU, and 128 GB RAM. We employed PyTorch 2.1.2 as our deep learning framework, CUDA version 12.1, and Ultralytics version 8.2.35 with Python 3.9.19. For instance segmentation training, AdamW was used as the optimizer and the hyperparameters were set as follows: learning rate = 0.01 for pre-training and 0.002 for fine-tuning; batch size = 16; epoch = 200; momentum = 0.937. The Distribution Focal Loss layer in the segmentation head was frozen for transfer learning to prevent overfitting.

### 3.3. Testing and Assessment

Table 2 presents the testing results of six models: Mask R-CNN, Mask R-CNN-SE [39], YOLACT, SOLOv2, YOLOv8-seg and YOLOv8-seg-CBAM-SE. All the models were trained on the same training set and reported their results on the same testing set from the base dataset. The results reveal that YOLOv8-seg-based models achieve superior performance over Mask R-CNN-based models in both recognition accuracy and boundary segmentation fidelity. In particular, YOLOv8-seg-CBAM-SE exhibits a 1.31% enhancement in IoU and an 8.79% improvement in mAP50, while delivering a 5-fold increase in inference speed (“Time” in Table 2) when compared to Mask R-CNN-SE. Performance comparisons were also conducted using the on-site dataset, as listed in Table 3. It can be seen that YOLOv8-seg-CBAM-SE achieves the highest mAP50 and mAP50-95 of 0.961 and 0.857, respectively. These remarkable enhancements in the accuracy and speed underscore the efficacy of integrating CBAM and SE attention mechanisms, which optimize feature representation without imposing additional computational overhead.

**Figure 16:**
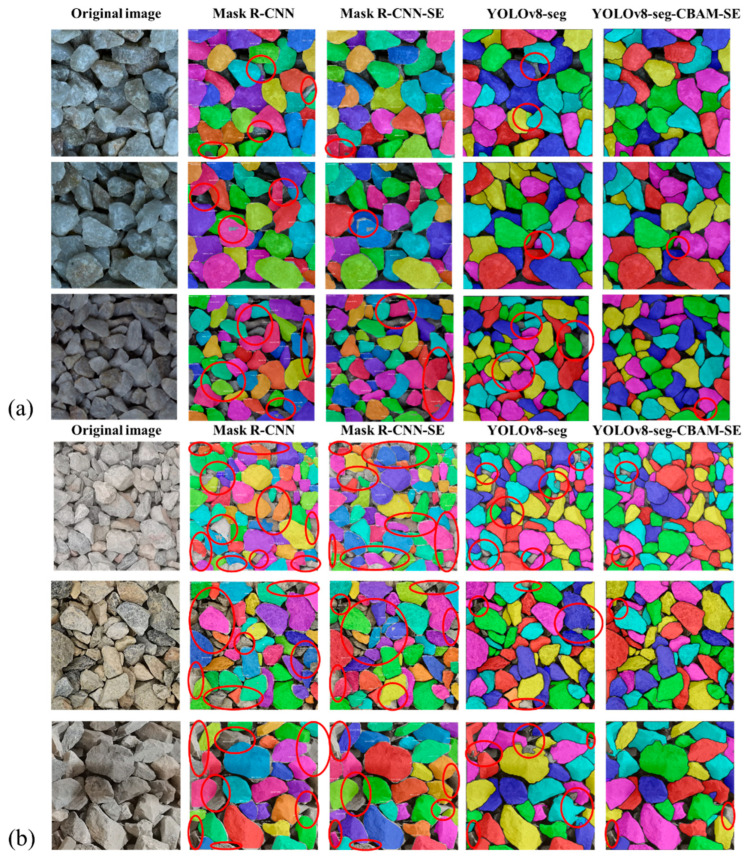
Segmentation results of the four models tested by the (**a**) base dataset; (**b**) on-site dataset.

Figure 16 depicts the segmentation outcomes of the four models. It is illustrated that Mask R-CNN-based modes exhibit limitations in contour segmentation, with significant occurrences of segmentation errors and omissions (red circle). Though YOLOv8-seg has a better segmentation performance, it still faces challenges in areas with unclear boundaries. In contrast, YOLOv8-seg-CBAM-SE is proven to obtain good instance segmentation results when compared to other models. One can observe that it not only can handle particle images from the well-trained base dataset, as shown in Figure 16a, but the images from the on-site dataset can also be segmented well, as shown in Figure 16b. Therefore, YOLOv8-seg-CBAM-SE can extract information such as counting, particle shape, color and texture, even when the particle boundaries are complex.

To assess the on-site applicability of the proposed model, we compared the four models in terms of parameter count, gradient count, per-epoch training time, and batch size, as shown in Table 4. Experimental results show that the YOLOv8-seg-CBAM-SE model exhibits an average per-epoch training time of 4.07 s. Under the same 10 GB GPU memory constraint, this model can process 16 images per batch during training, demonstrating its efficient resource utilization.

**Figure 17:**
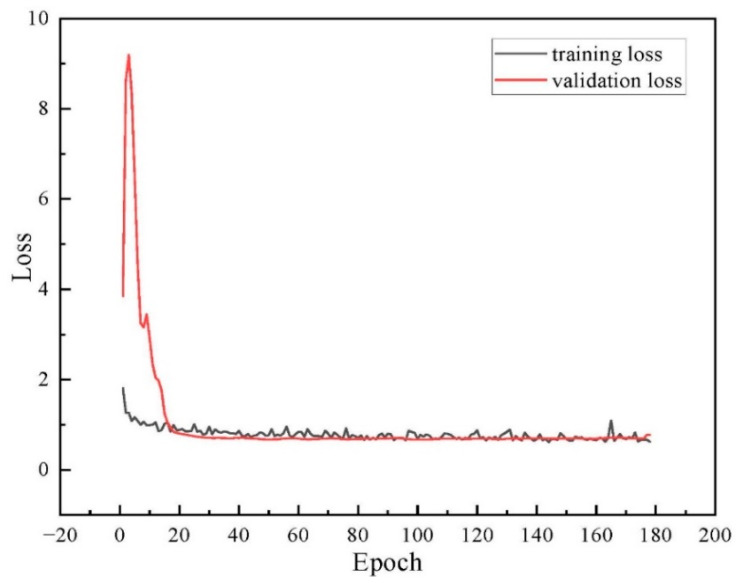
The training and validation loss curve for the on-site dataset.

Figure 17 depicts the training and validation loss curves of YOLOv8-seg-CBAM-SE for the on-site dataset. It indicates that the model tends to converge when iterating to the 150th epoch. The training process was terminated at the 177th epoch based on the early stopping criterion with a patience value of 50 epochs. As a result, the total training time is less than 10 min. These findings suggest that our model can achieve high operational efficiency in on-site scenarios.

## 4. Experiments and Results

### 4.1. Testing Material

**Figure 18:**
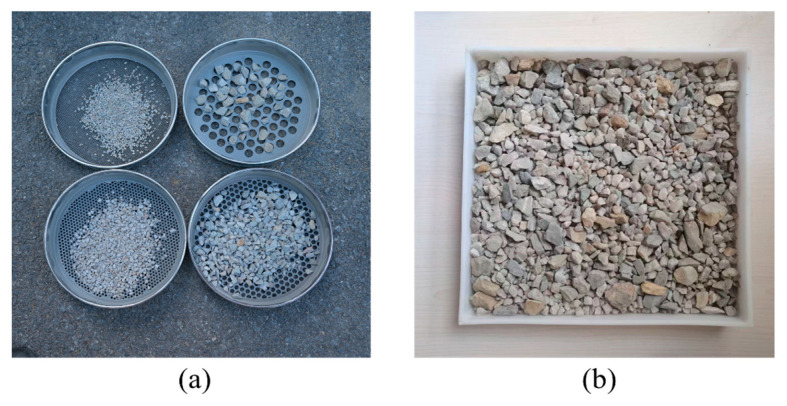
Sample of rockfill for gradation detection: (**a**) mechanical sieving; (**b**) image acquisition.

Rockfill with a size range from 2 mm to 25 mm was collected as a representative for granular materials. A standard screening experiment was first conducted to quantify the gradations by using sieves with sizes of 2, 5, 10 and 20 mm, as shown in Figure 18a. Subsequently, the rockfill was mixed and placed on the tray, and images were captured by means of smartphone photography, as shown in Figure 18b.

### 4.2. Particle Segmentation

**Figure 19:**
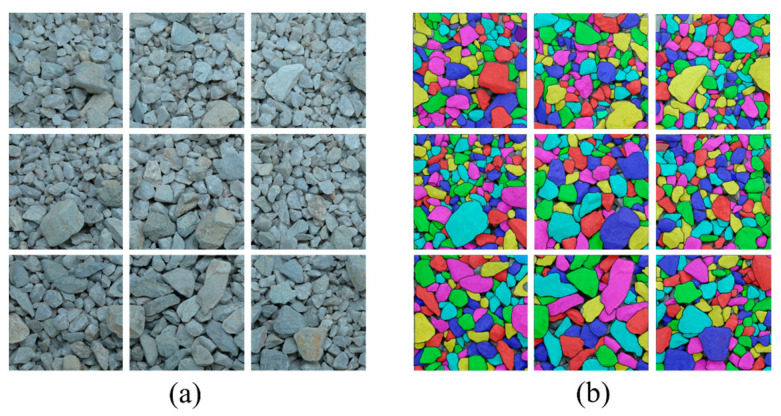
Sample images and the corresponding segmentation results: (**a**) testing sets; (**b**) segmentation results.

The resolution of the original image was 3200 × 3200. After cropping and labeling, 9 sub-images with a resolution of 640 × 640 were used as the testing sets, as shown in Figure 19a. Since the type of particles in the current set is consistent with that in the on-site dataset, no more model fine-tuning is needed here. Figure 19b presents the segmentation results obtained using the YOLOv8-seg-CBAM-SE model. It can be observed that the model performs well in handling adhered and overlapped particles with varying sizes, demonstrating a high accuracy in segmenting particle contours, capturing fine details and boundaries effectively.

### 4.3. Gradation Calculation

**Figure 20:**
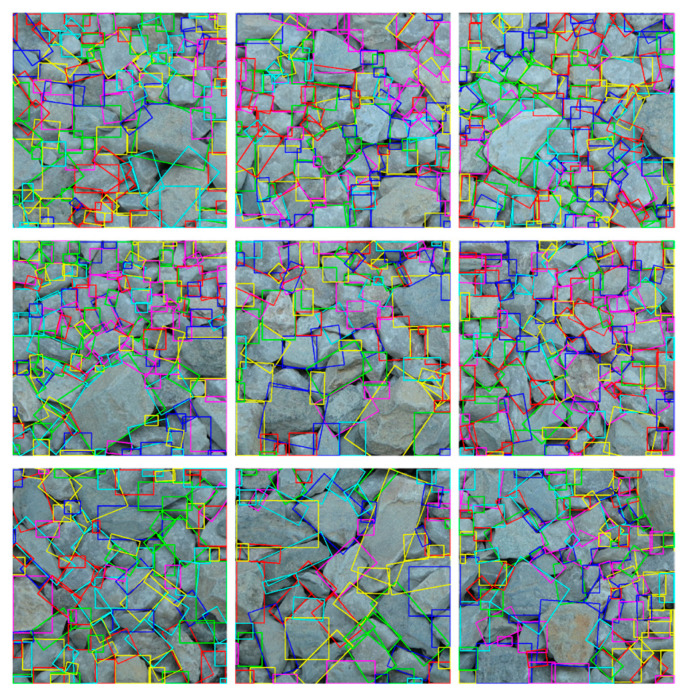
Minimum Area Rectangle of particles.

The MAR of each particle contour, derived from the segmentation process, is used to quantify the elongation and shape characteristics of particles, as illustrated in Figure 20. Two errors indices, the MAE and the root mean square error (RMSE), are calculated to quantitatively assess the discrepancies between the proposed method and mechanical sieving, as follows:

(21) $$\mathrm{MAE}=\frac{1}{n}\sum_{i=1}^{n}|y_{i}-x_{i}|,$$

(22) $$\mathrm{RMSE}=\sqrt{\frac{1}{n}\sum_{i=1}^{n}{\left(y_{i}-x_{i}\right)}^{2}}$$

where n denotes the number of sieves; i denotes the ith sieve; x_i_ and y_i_ denote the PGD result of the IIS-based method and machine sieving, respectively.

**Figure 21:**
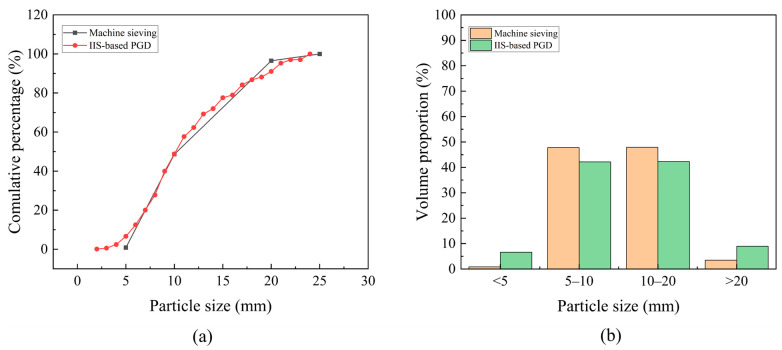
Comparison of PGD results obtained by IIS-based PGD and mechanical sieving: (**a**) gradation curves; (**b**) volume proportion.

The results show that MAE equals 2.843% and RMSE equals 3.973%. Figure 21 presents the gradation curves and gradation proportions obtained by both IIS-based PGD and mechanical sieving. Notably, the PGD results derived by the proposed method exhibit strong alignment with mechanical sieving measurements, demonstrating an excellent capability to maintain high accuracy and efficiency in gradation detection under overlapped particle conditions.

## 5. Discussion

### 5.1. Shooting Parameter Constraints

The proposed method of this paper is suitable for rapid field assessment of particle size distribution (PSD) in earth–rock dams. Shooting distance, shooting angle and hardware requirements are shown in Table 5. A camera-to-subject distance range of 40–80 cm was maintained during imaging to yield viable particle counts per segmented tile. For larger particle sizes, the shooting distance proportionally increased. Imaging devices were required to exceed a 3000 × 3000-pixel resolution. To mitigate occlusion-driven segmentation errors resulting from excessive perspective angles, the shooting angle was constrained to ≤20° relative to the specimen plane.

### 5.2. Impact of Transfer Learning

**Figure 22:**
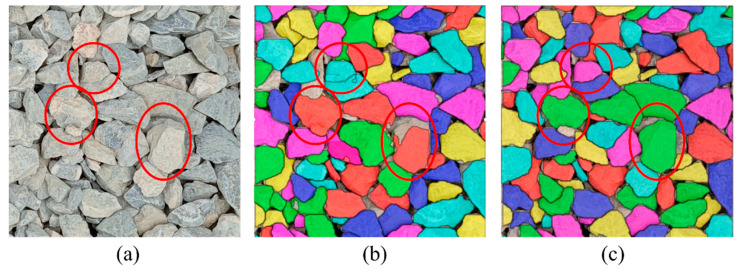
Comparison of the segmentation result without and with transfer learning: (**a**) on-site imagery; (**b**) segmentation result without transfer learning; (**c**) segmentation result with transfer learning.

To evaluate the impact of transfer learning on the robustness and generalization ability of YOLOv8-seg-CBAM-SE, this section conducted testing on densely stacked particle image segmentation with and without transfer learning. Figure 22a shows a particle image captured in the field, which has substantial morphological, color, and brightness heterogeneity when compared to the base dataset. Figure 22b,c illustrate the segmentation results with and without transfer learning.

It can be found that the model without transfer learning achieves competent segmentation on a subset of particles but also produces notable segmentation errors, such as boundary discontinuities, as shown by the red circles in Figure 22b. These errors critically compromise its utility for precision-dependent PGD tasks. The model with transfer learning weights demonstrates a significant improvement in segmentation performance, as shown in Figure 22c. Table 6 presents the metrics and segmentation results, and confirms that transfer learning not only enhances the model’s generalization ability but also addresses the limitation of IIS-based PGD methods in the segmentation of different particle types.

**Figure 23:**
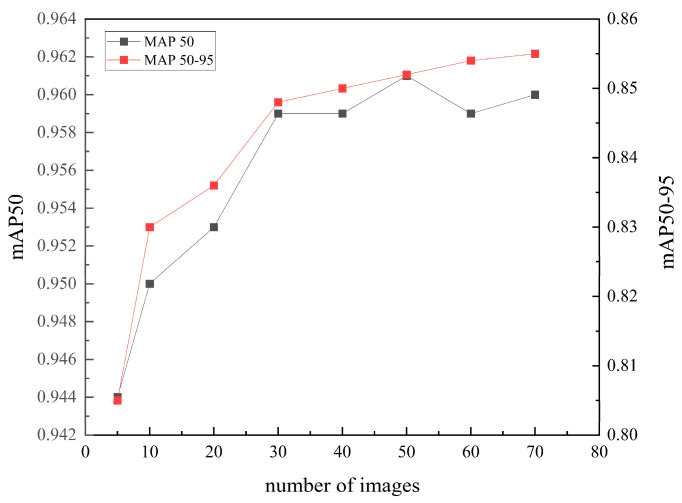
Relationship between the number of images in the on-site dataset and the two metrics: mAP50 and mAP50-95.

We also analyze the relationship between the number of images in the on-site dataset and the corresponding mAP50 and mAP50-95 metrics, as illustrated in Figure 23. It can be found that both mAP50 and mAP50-95 exhibit a notable upward trend as the number of images in the on-site dataset increases. This indicates that the model’s segmentation accuracy and robustness improve with the availability of more training data. Meanwhile, as the dataset size expands, the improvement in metrics begins to plateau, demonstrating diminishing returns with additional data. Notably, the segmentation performance achieves a plateau (mAP50: 96.1%; mAP50-95: 85.7%) with merely 50 training images, which means that only 2–3 standard smartphone-captured images (typically exceeding 3000 × 3000 pixels) should be processed for PGD. These findings indicate that the proposed YOLOv8-seg-CBAM-SE model uses transfer learning. It achieved high segmentation accuracy with minimal data. This approach substantially reduces the computational and time overhead typically required by deep learning in PGD applications.

### 5.3. On-Site Implementation Procedure

To validate the field applicability of our method, this section elaborates on its operational workflow under actual on-site conditions:

1. Image Acquisition and Pre-processing: A limited number of on-site images were acquired at construction sites and pre-processed using the methodology proposed in this paper to establish the transfer learning dataset.

2. Model Weight Fine-tuning: Based on the transfer learning dataset mentioned above, the pre-trained weights were fine-tuned through transfer learning to obtain model segmentation weights tailored for local particle segmentation.

3. Instance Segmentation and Gradation Analysis: On-site images from different zones of the rockfill dam site were subjected to instance segmentation to obtain granular contour data. Gradation detection for each zone was subsequently calculated utilizing the PGD method proposed in this work.

## 6. Conclusions

In this study, we introduce an on-site PGD method that combines mobile-based photography with an enhanced YOLOv8-seg model. This solution addresses the shortcomings of traditional approaches, contributing to automation in construction workflows, cutting labor costs, minimizing structural disruption, and offering an efficient framework for accurate particle size distribution detection in earth–rockfill dam construction. Addressing the shortcomings of traditional approaches, this solution enhances automation in construction workflows. As a result, it cuts labor costs and minimizes structural disruption. It also provides an efficient framework for accurate particle size distribution detection in earth–rockfill dam construction. The key findings of this investigation can be summarized as follows:

(1) A dual-attention YOLOv8-seg-CBAM-SE network was constructed by introducing the CBAM and SE block into the original YOLOv8-seg framework to accurately segment densely stacked particles. This integration synergistically improves feature representation and discrimination capabilities while preserving model simplicity and real-time performance.

(2) The network was first pre-trained by using a basic dataset of laboratory-captured images, and then followed by refinement through transfer learning with a limited set of on-site images to optimize weight parameters. The results indicate that YOLOv8-seg-CBAM-SE achieves a mAP50 of 97.8% for the base dataset and 96.1% for the on-site dataset, realizing a high-precision instant segmentation of stacked particles with multi-scale sizes when compared to other networks.

(3) Coordinate transformations were implemented to map pixel coordinates to world coordinates, effectively addressing geometric distortions caused by capturing angle variations and lens-induced aberrations. This ensures accurate spatial representation and enhances the fidelity of world coordinate reconstruction. By correcting distortions and aligning image data with physical dimensions, the method significantly improves the reliability of particle gradation calculations.

(4) The MAR algorithm incorporating a revolving angle strategy was employed to calculate the particle gradation. By performing angular discretization within a predefined rotational range, the method computes geometric projections at incremental angles and identifies the optimal MAR through comparative evaluation of enclosed areas. The precision of this approach ensures accurate particle size distribution analysis, making it a reliable alternative to traditional manual methods.

(5) This study also highlights the critical role of transfer learning in overcoming the limitations of IIS-based PGD methods. By refining model weights, transfer learning significantly improves segmentation accuracy and generalization ability, enabling precise analysis of different particle types.

Limitations: External factors such as debris and dust in practical engineering scenarios may compromise the precision of instance segmentation. GPU memory exceeding 8 GB for model training was required. In future work, we will further consider more complex on-site shooting conditions, including uneven light intensity, dust pollution that changes the color and texture of particles, interference from construction materials, etc. These factors will all affect the imaging quality and increase the difficulty of image instant segmentation. All these challenges will be addressed through the combination of parameter optimization and morphological pre-processing techniques in segmentation to ensure the robustness of the model. Additionally, we will enhance the basic dataset by incorporating particle data of a wider range of rock types and under different natural conditions to improve the generalization ability of the model.

## Figures and Tables

**Table 1:** Numbers of images for model pre-training.

Data Set	Numbers of Images		
	Group I (Figure 14a)	Group II (Figure 14b)	Group III (Figure 14c)
Training set	355	559	1780
Validation set	104	201	446
Testing set	-	-	560

**Table 2:** Performance comparisons of the models tested for the base dataset.

Model	Precision	Recall	IoU	mAP50	mAP50-95	Time (s)
Mask R-CNN	-	-	0.915	0.870	-	1.57
Mask R-CNN-SE	-	-	0.916	0.899	-	1.81
YOLACT	-	-	0.720	0.591	0.178	0.26
SOLOv2	-	-	0.930	0.955	0.851	0.39
YOLOv8-seg	0.915	0.934	0.924	0.975	0.913	0.35
YOLOv8-seg-CBAM-SE	0.923	0.935	0.928	0.978	0.917	0.36

**Table 3:** Performance comparisons of the models tested for the on-site dataset.

Model	Precision	Recall	IoU	mAP50	mAP50-95	Time (s)
Mask R-CNN	-	-	0.883	0.773	-	2.50
Mask R-CNN-SE	-	-	0.897	0.805	-	2.75
YOLACT	-	-	0.678	0.417	0.109	0.36
SOLOv2	-	-	0.920	0.935	0.809	0.50
YOLOv8-seg	0.885	0.896	0.902	0.955	0.803	0.45
YOLOv8-seg-CBAM-SE	0.905	0.899	0.910	0.961	0.857	0.44

**Table 4:** Performance comparisons of the six models.

Model	Parameters	Gradients	Training Time (s)	Batch Size
Mask R-CNN	44M	44M	507	4
Mask R-CNN-SE	44M	44M	508	4
YOLACT	30.74M	30.74M	40	4
SOLOv2	65.22M	65.22M	47	4
YOLOv8-seg	3.26M	3.26M	4.05	16
YOLOv8-seg-CBAM-SE	3.28M	3.28M	4.07	16

**Table 5:** Shooting requirements.

Shooting Distance	Shooting Angle	Imaging Device Pixel Requirement
40 cm–80 cm	≤20°	>3000 × 3000 pixels

**Table 6:** Performance comparison of the model with and without transfer learning.

Weight	IoU	mAP50	mAP50-95
Pre-trained weight	0.903	0.939	0.805
Fine-tuned weight	0.910	0.961	0.857

## Data Availability

The datasets from this study are available upon request to the corresponding author.

## Abbreviations

The following abbreviations are used in this manuscript:

PGD	Particle gradation detection
CV	Computer vision
DIP	Digital image processing
DL	Deep learning
IIS	Image instance segmentation
YOLO	You Only Look Once
SE	Squeeze and excitation
MAR	Minimum area rectangle
GIS	Geographic information system
SNR	Signal-to-noise ratio
MAE	Mean absolute error
ISAT-SAM	Interactive Segment Anything Tool–Segment Anything Model
CBAM	Convolutional block attention module
P	Precision
R	Recall
mAP	Mean average precision
TP	True positives
FP	False positives
FN	False negatives
IoU	Intersection over union
PR	Precision–recall
AABB	Axis-aligned bounding box
RMSE	Root mean square error

## References

[1] Wu J., Feng M., Mao X., Xu J., Zhang W., Ni X., Han G. (2018). Particle Size Distribution of Aggregate Effects on Mechanical and Structural Properties of Cemented Rockfill: Experiments and Modeling. Constr. Build. Mater..

[2] Meng M., Deng S., Cui H., Yuan Z., He X. (2024). Impact of Initial Gradation on Compaction Characteristics and Particle Crushing Behavior of Gravel under Dynamic Loading. Powder Technol..

[3] Liu D., Yang J., Dong B. (2022). Discrete Element Analysis of the Influence of Compaction Quality on Mechanical Properties of Rockfill Materials. Comput. Geotech..

[4] Wang S., Miao Y., Wang L. (2023). Effect of Grain Size Composition on Mechanical Performance Requirement for Particles in Aggregate Blend Based on Photoelastic Method. Constr. Build. Mater..

[5] Zhu S., Zhong C.-X., Zheng X.-L., Gao Z.-P., Zhan Z.-G. (2018). Filling Standards and Gradation Optimization of Rockfill Materials. Chin. J. Geotech. Eng..

[6] Cui W., Liu G., Song H., Wang C. (2023). Mesoscopic Analysis of the Compaction Characteristics of Rockfill Materials Considering Gradation and Shape. Int. J. Geomech..

[7] Liu D., Li Z., Lian Z. (2014). Compaction Quality Assessment of Earth-Rock Dam Materials Using Roller-Integrated Compaction Monitoring Technology. Autom. Constr..

[8] Chen H., Kang A.-H., Li B., Zhang Y., Zhu Z. (2025). Research on Skid Resistance and Prediction Model of Hot In-Place Recycled Asphalt Pavement Based on Gradation and Aggregate Characteristics. Constr. Build. Mater..

[9] Roostaei M., Hosseini S.A., Soroush M., Velayati A., Alkouh A., Mahmoudi M., Ghalambor A., Fattahpour V. (2020). Comparison of Various Particle-Size Distribution-Measurement Methods. SPE Reserv. Eval. Eng..

[10] Zhou Y., Zhou H., Chen T., Hu C., Liang Z., Zhao C., Wang F. (2023). Detection of Rockfill Gradation Based on Video Image Recognition. Autom. Constr..

[11] Igathinathane C., Pordesimo L.O., Columbus E.P., Batchelor W.D., Sokhansanj S. (2009). Sieveless Particle Size Distribution Analysis of Particulate Materials through Computer Vision. Comput. Electron. Agric..

[12] Yang B., Zhang X., Chen L., Yang H., Gao Z. (2017). Edge Guided Salient Object Detection. Neurocomputing.

[13] Bai F., Fan M., Yang H., Dong L. (2021). Image Segmentation Method for Coal Particle Size Distribution Analysis. Particuology.

[14] Yan K., Liu G., Li Q., Jiang C., Ren T., Li Z., Xie L., Wang L. (2024). Corrosion Characteristics and Evaluation of Galvanized High-Strength Steel Wire for Bridge Cables Based on 3D Laser Scanning and Image Recognition. Constr. Build. Mater..

[15] Amiriebrahimabadi M., Rouhi Z., Mansouri N. (2024). A Comprehensive Survey of Multi-Level Thresholding Segmentation Methods for Image Processing. Arch. Comput. Methods Eng..

[16] Yang M., Ding J., Li W., Tian A., Pei L., Hao X. (2023). A Coarse Aggregate Gradation Detection Method Based on 3D Point Cloud. Constr. Build. Mater..

[17] Guo Q., Wang Y., Yang S., Xiang Z. (2022). A Method of Blasted Rock Image Segmentation Based on Improved Watershed Algorithm. Sci. Rep..

[18] Ren Z., Tan Y., Huang L., Yu H. (2022). Optimization of Automatic Extraction Procedure for Particles in Asphalt Mixture towards Superior Robustness and Accuracy. Constr. Build. Mater..

[19] Sun Q., Zheng J., Li C. (2019). Improved Watershed Analysis for Segmenting Contacting Particles of Coarse Granular Soils in Volumetric Images. Powder Technol..

[20] Guo Q., Bian Y., Li L., Jiao Y., Tao J., Xiang C. (2015). Stereological Estimation of Aggregate Gradation Using Digital Image of Asphalt Mixture. Constr. Build. Mater..

[21] Yu S., Wen Y., Chen Z., Zhang G., Wang Y., Hao J., Zhang Q. (2021). A Rapid Gradation Detection System for Earth and Stone Materials Based on Digital Image. Adv. Civ. Eng..

[22] Wang W., Li C., Zhao X., Song P., Li J., Zheng J., Bu S., Wang X. (2024). An Image-Based Method for Evaluating Changes in Particle Size and Morphology Distributions of Aggregate Materials after Vibratory Compaction Test. Constr. Build. Mater..

[23] Liang Z., Nie Z., An A., Gong J., Wang X. (2019). A Particle Shape Extraction and Evaluation Method Using a Deep Convolutional Neural Network and Digital Image Processing. Powder Technol..

[24] Wang W., Su C., Zhang H. (2022). Automatic Segmentation of Concrete Aggregate Using Convolutional Neural Network. Autom. Constr..

[25] Tsalicoglou C., Rösgen T. (2022). Deep Learning Based Instance Segmentation of Particle Streaks and Tufts. Meas. Sci. Technol..

[26] Hu X., Fang H., Yang J., Fan L., Lin W., Li J. (2022). Online Measurement and Segmentation Algorithm of Coarse Aggregate Based on Deep Learning and Experimental Comparison. Constr. Build. Mater..

[27] Li F., Liu X., Yin Y., Li Z. (2024). A Novel Method for Particle Instance Segmentation and Size Measurement. IEEE Trans. Instrum. Meas..

[28] Chen N., Ma X., Luo H., Peng J., Jin S., Wu X., Zhou Y. (2024). Stone Segmentation Based on Improved U-Net Network. Signal Image Video Process..

[29] Wei X., Zhao Y., Lu X., Zhang M., Du J., Guo X., Zhao C. (2024). A High-Throughput Method for Monitoring Growth of Lettuce Seedlings in Greenhouses Based on Enhanced Mask2Former. Comput. Electron. Agric..

[30] He K., Gkioxari G., Dollár P., Girshick R. Mask R-Cnn. Proceedings of the IEEE International Conference on Computer Vision.

[31] Peng C., Zheng L., Liang Q., Li T., Wu J., Cheng X. (2024). ST-SOLOv2: Tracing Depth Hoar Layers in Antarctic Ice Sheet From Airborne Radar Echograms with Deep Learning. IEEE Trans. Geosci. Remote Sens..

[32] Shamsollahi D., Moselhi O., Khorasani K. (2024). Automated Detection and Segmentation of Mechanical, Electrical, and Plumbing Components in Indoor Environments by Using the YOLACT++ Architecture. J. Constr. Eng. Manag..

[33] Bi X., Hu J., Xiao B., Li W., Gao X. (2023). IEMask R-CNN: Information-Enhanced Mask R-CNN. IEEE Trans. Big Data.

[34] Lai W., Hu F., Kong X., Yan P., Bian K., Dai X. (2022). The Study of Coal Gangue Segmentation for Location and Shape Predicts Based on Multispectral and Improved Mask R-CNN. Powder Technol..

[35] Zhang R., Li K., Yu F., Zhang H., Gao Z., Huang Y. (2023). Aggregate Particle Identification and Gradation Analysis Method Based on the Deep Learning Network of Mask R-CNN. Mater. Today Commun..

[36] Qin J., Wang J., Lei T., Sun G., Yue J., Wang W., Chen J., Qian G. (2023). Deep Learning-Based Software and Hardware Framework for a Noncontact Inspection Platform for Aggregate Grading. Measurement.

[37] Zhang Z., Yin X., Yan Z. (2022). Rapid Data Annotation for Sand-like Granular Instance Segmentation Using Mask-RCNN. Autom. Constr..

[38] Li X., Li S., Dong L., Su S., Hu X., Lu Z. (2023). An Image Segmentation Method of Pulverized Coal for Particle Size Analysis. Int. J. Min. Sci. Technol..

[39] Redmon J., Divvala S., Girshick R., Farhadi A. You Only Look Once: Unified, Real-Time Object Detection. Proceedings of the 2016 IEEE Conference on Computer Vision and Pattern Recognition (CVPR).

[40] Hussain M. (2024). YOLOv1 to v8: Unveiling Each Variant–A Comprehensive Review of YOLO. IEEE Access.

[41] Badgujar C.M., Poulose A., Gan H. (2024). Agricultural Object Detection with You Only Look Once (YOLO) Algorithm: A Bibliometric and Systematic Literature Review. Comput. Electron. Agric..

[42] Ali M.L., Zhang Z. (2024). The YOLO Framework: A Comprehensive Review of Evolution, Applications, and Benchmarks in Object Detection. Computers.

[43] Li X., Zhao S., Chen C., Cui H., Li D., Zhao R. (2024). YOLO-FD: An Accurate Fish Disease Detection Method Based on Multi-Task Learning. Expert. Syst. Appl..

[44] Ragab M.G., Abdulkadir S.J., Muneer A., Alqushaibi A., Sumiea E.H., Qureshi R., Al-Selwi S.M., Alhussian H. (2024). A Comprehensive Systematic Review of YOLO for Medical Object. Detection (2018 to 2023). IEEE Access.

[45] Kang S., Hu Z., Liu L., Zhang K., Cao Z. (2025). Object Detection YOLO Algorithms and Their Industrial Applications: Overview and Comparative Analysis. Electronics.

[46] Mao M., Hong M. (2025). YOLO Object Detection for Real-Time Fabric Defect Inspection in the Textile Industry: A Review of YOLOv1 to YOLOv11. Sensors.

[47] Chen D., Kang F., Li J., Zhu S., Liang X. (2024). Enhancement of Underwater Dam Crack Images Using Multi-Feature Fusion. Autom. Constr..

[48] Zhang B., Li J., Bai Y., Jiang Q., Yan B., Wang Z. (2023). An Improved Microaneurysm Detection Model Based on SwinIR and YOLOv8. Bioengineering.

[49] Zhang X., Dong T., Yan L., Yang Z., Zhang J. LAtt-Yolov8-Seg: Video Real-Time Instance Segmentation for Urban Street Scenes Based on Focused Linear Attention Mechanism. Proceedings of the International Conference on Computer Vision and Deep Learning.

[50] Wang P., Deng H., Guo J., Ji S., Meng D., Bao J., Zuo P. (2024). Leaf Segmentation Using Modified YOLOv8-Seg Models. Life.

[51] Xiong C., Zayed T., Abdelkader E.M. (2024). A Novel YOLOv8-GAM-Wise-IoU Model for Automated Detection of Bridge Surface Cracks. Constr. Build. Mater..

[52] Meng A., Zhang X., Yu X., Jia L., Sun Z., Guo L., Yang H. (2024). Investigation on Lightweight Identification Method for Pavement Cracks. Constr. Build. Mater..

[53] Xiao B., Xiao H., Wang J., Chen Y. (2022). Vision-Based Method for Tracking Workers by Integrating Deep Learning Instance Segmentation in off-Site Construction. Autom. Constr..

[54] Bai R., Wang M., Zhang Z., Lu J., Shen F. (2023). Automated Construction Site Monitoring Based on Improved YOLOv8-Seg Instance Segmentation Algorithm. IEEE Access.

[55] Hu Y., Wang J., Wang X., Sun Y., Yu H., Zhang J. (2024). Real-Time Evaluation of the Blending Uniformity of Industrially Produced Gravelly Soil Based on Cond-YOLOv8-Seg. J. Ind. Inf. Integr..

[56] Dan H.-C., Huang Z., Lu B., Li M. (2024). Image-Driven Prediction System: Automatic Extraction of Aggregate Gradation of Pavement Core Samples Integrating Deep Learning and Interactive Image Processing Framework. Constr. Build. Mater..

[57] Hu Y., Wang J., Wang X., Guan T. (2023). Occlusion-Aware Particle Size Distribution Detection of Gravel Material Based on the Improved Bilayer Convolutional Network. Constr. Build. Mater..

[58] Cheng X., Yang J., Fang H., Yu W. (2025). Research on the Online Prediction Method of Coarse Aggregates Packing Void Ratio Based on Particle Shape and Grading. Constr. Build. Mater..

[59] Chiu M.-C., Tsai H.-Y., Chiu J.-E. (2022). A Novel Directional Object Detection Method for Piled Objects Using a Hybrid Region-Based Convolutional Neural Network. Adv. Eng. Inform..

[60] Xia X., Meng Z., Han X., Li H., Tsukiji T., Xu R., Zheng Z., Ma J. (2023). An Automated Driving Systems Data Acquisition and Analytics Platform. Transp. Res. Part. C Emerg. Technol..

[61] Fan H., Tian Z., Xu X., Sun X., Ma Y., Liu H., Lu H. (2022). Rockfill Material Segmentation and Gradation Calculation Based on Deep Learning. Case Stud. Constr. Mater..

[62] Zhang D. An Extended Opening Operation and Its Application in Image Processing. Proceedings of the 2008 International Conference on MultiMedia and Information Technology.

[63] Chen G., Cui G., Jin Z., Wu F., Chen X. (2019). Accurate Intrinsic and Extrinsic Calibration of RGB-D Cameras with GP-Based Depth Correction. IEEE Sens. J..

[64] Woo S., Park J., Lee J.-Y., Kweon I.S. Cbam: Convolutional Block Attention Module. Proceedings of the European Conference on Computer vision (ECCV).

[65] Hu J., Shen L., Sun G. Squeeze-and-Excitation Networks. Proceedings of the IEEE Conference on Computer Vision and Pattern Recognition.

[66] Toussaint G.T. Solving Geometric Problems with the Rotating Calipers. Proceedings of the IEEE Melecon.

[67] Li Q., Dai G., Wang M. (2003). Research on Algorithm for Generating Min-Area Rectangle Encasing Box for Closed Contour. J. Earth Sci..

